# Gender differences in older adults’ romantic relationship preference and sexual satisfaction

**DOI:** 10.1093/geroni/igaf122.2772

**Published:** 2025-12-31

**Authors:** William Henninger, Melinda Heinz, Nathan Taylor

**Affiliations:** University of Northern Iowa, Cedar Falls, Iowa, United States; University of Northern Iowa, Cedar Falls, Iowa, United States; University of Northern Iowa, Cedar Falls, Iowa, United States

## Abstract

Socioemotional Selectivity Theory posits that as people age, they prioritize immediate emotional satisfaction over long-term goals due to the perception of limited time. This shift influences older adults’ romantic relationships, increasing the likelihood of seeking meaningful connections and avoiding negative emotions. With healthy relationships linked to physical, psychological, and financial benefits, research is needed to further understand older adult romantic relationships. The purpose of this study is to explore gender differences in older adults’ preferences for long-term and short-term relationships and their sexual satisfaction. Older adults (n = 155) aged 65-95 years completed surveys assessing short-term and long-term relationship preferences, current positive and negative sexual satisfaction, and predicted future positive and negative sexual satisfaction. Results indicate that males report higher preferences for long-term relationships (t(153) = 3.86, p < .001) and predicted more positive future sexual satisfaction (t(89) = 3.01, p < .01). Females were nearing significance of more negative current sexual satisfaction (t(52) = -1.57, p = .06). As the aging population increases and gray divorce is more prevalent, these findings have important implications for researchers, practitioners, and aging adults. Differences in relationship preference may result in disconnect between potential partners due to the type of relationship desired. Additionally, predicting more positive future sexual satisfaction may create expectations that could result in relationship distress and dissatisfaction. Future research is needed to explore the dyadic effects of differences in relationship and sexual preferences. Additionally, more diverse samples such as with same sex relationships and the oldest old are needed.

